# Hydrophilicity Affecting the Enzyme-Driven Degradation of Piezoelectric Poly-l-Lactide Films

**DOI:** 10.3390/polym13111719

**Published:** 2021-05-24

**Authors:** Lea Gazvoda, Bojana Višić, Matjaž Spreitzer, Marija Vukomanović

**Affiliations:** 1 Advanced Materials Department, Jožef Stefan Institute, 1000 Ljubljana, Slovenia; lea.udovc@ijs.si (L.G.); matjaz.spreitzer@ijs.si (M.S.); 2 Jožef Stefan International Postgraduate School, 1000 Ljubljana, Slovenia; 3 Condensed Matter Physics Department, Jožef Stefan Institute, 1000 Ljubljana, Slovenia; bojana.visic@ijs.si; 4 Institute of Physics Belgrade, University of Belgrade, Pregrevica 118, 11080 Belgrade, Serbia

**Keywords:** poly-l-lactic acid, enzymatically catalyzed degradation, piezoelectricity, bulk erosion, surface erosion

## Abstract

Biocompatible and biodegradable poly-l-lactic acid (PLLA) processed into piezoelectric structures has good potential for use in medical applications, particularly for promoting cellular growth during electrostimulation. Significant advantages like closer contacts between cells and films are predicted when their surfaces are modified to make them more hydrophilic. However, there is an open question about whether the surface modification will affect the degradation process and how the films will be changed as a result. For the first time, we demonstrate that improving the polymer surface’s wettability affects the position of enzyme-driven degradation. Although it is generally considered that proteinase K degrades only the polymer surface, we observed the enzyme’s ability to induce both surface and bulk degradation. In hydrophilic films, degradation occurs at the surface, inducing surface erosion, while for hydrophobic films, it is located inside the films, inducing bulk erosion. Accordingly, changes in the structural, morphological, mechanical, thermal and wetting properties of the film resulting from degradation vary, depending on the film’s wettability. Most importantly, the degradation is gradual, so the mechanical and piezoelectric properties are retained during the degradation.

## 1. Introduction

Poly-l-lactic acid (PLLA), a biosynthetic thermoplastic polyester, is widely used in the biomedical field because it is biocompatible and biodegradable [1,2]. It can also be formed into different shapes (2D coatings and films, micro- and nano-powders, 3D scaffolds, etc.) with different properties, depending on the requirements [3]. Accordingly, PLLA is used in drug-delivery systems [4], tissue engineering [5], wound dressing [6], implantation [7] and many more [8,9].

A very poorly explored and particularly important aspect of PLLA’s application in biomedicine is associated with the possibility of processing it into a piezoelectric material. Due to its helix structure, PLLA exhibits shear piezoelectricity [10]. When PLLA is oriented and crystalline, usually as fibers or films, the chains are strained, the molecular dipoles are aligned, and a voltage difference is observed [11]. Such a structure, formulated as a piezoelectric scaffold, can be applied for piezo-stimulation and promoting cell proliferation. The low piezoelectric effect of PLLA (compared to piezoceramics) is comparable to the piezoelectricity of natural biomacromolecules (i.e., collagen) [12] and can be efficient enough to relate with biological systems [13]. It makes this polymer particularly appropriate for regenerative medical use to accelerate the wound-healing process. When applied for wound healing, the safe biodegradation of piezoelectric PLLA films is very important. The process needs to be steady, and the biodegradation (followed by a change of mechanical, structural, morphological and piezoelectric properties) should follow the kinetics of the tissue regeneration so that at the end, no material residues are left in the system when the new tissue is formed.

When PLLA is in contact with biological media, cleavage of the ester bonds occurs in the bulk matter, usually by hydrolysis, into lactic acid, carbon dioxide and water [14]. Under in vivo conditions, due to the inflammation process during the injury, the degradation process is enhanced by the enzymes present that degrade the polymer matrix [1]. Since PLLA is more hydrophobic, swelling of the polymer film occurs with the diffusion of water inside the polymer bulk, which triggers the degradation inside the material [14]. However, when enzymes are present, it is generally considered that surface degradation is favorable since the diffusion of enzymes inside the film is aggravated [15]. Proteinase K catalyzes the degradation of PLLA several times faster in amorphous than in crystalline regions [16]. At the surface, degradation is limited to just the amorphous parts [15]. Crystalline residues, remaining after the degradation of the connected amorphous areas, are released into the surrounding liquid medium or accumulate on the surface and increase crystallinity [15]. Polymer crystallinity and chain orientation have been determined to have an important role in the process of enzyme-induced PLLA degradation. H. Tsuji et al. showed that the initially higher crystallinity of the PLLA films slows the degradation process and that the higher polymer-chain orientation also limits the enzyme intake into the bulk [15]. In addition, Rangari et al. observed that during the hydrolysis of the amorphous part on the surface in uniaxially prepared crystalline PLLA films, crystallinity plays the dominant role in determining the extent of the degradation, compared to the orientation of the polymer chains [16].

Understanding the degradation mechanism in biologically relevant surroundings is a key issue for using a piezoelectric polymer in biomedical applications. Although there is a detailed study of the structural changes that follow enzymatic degradation (change in crystallinity, chain orientation, chain mobility, etc.), very little knowledge is available on the change in the piezoelectric and mechanical properties of PLLA films during the degradation process. All the information regarding PLLA degradation is available for polymers with a hydrophobic surface. However, the hydrophobic surface is the main disadvantage of PLLA, potentially resulting in a low cell affinity and an inflammatory response [17]. A particular lack of information was observed for the case of the degradation of surface-modified PLLA hydrophilic films. With that in mind, we focused our investigations on two questions:

(i) How will the degradation be affected if the surface of the PLLA film is modified from hydrophobic to hydrophilic (more favorable to cells)?
(ii) What will happen to the mechanical and piezoelectric properties of the PLLA once the film is immersed in a liquid medium containing proteinase K (as an imitation of the inflammation response at the site of a wound)?

Therefore, in this study, we compare the enzymatically catalyzed degradation process of a uniaxial drawn piezoelectric PLLA film with and without surface modification, and with it the mechanical and piezoelectric changes that occur during the degradation process to achieve a more stable and gradual loss of piezoelectric properties during the process. Films with improved wettability should have greater potential for medical applications due to the proposed better affinity of the cells for the film.

## 2. Materials and Methods

### 2.1. Materials

Poly-l-lactic acid (PLLA) having a molecular weight with an approximate value of 150 kDa (Goodfellow, Cambridge, Ltd., UK), bovine serum albumin (BSA) (Fisher scientific, Leics, UK), methylene blue (MB) (Alfa Aesar, Thermo Fisher GmbH, Kandel, Germany) were used. Enzyme proteinase K was purchased from ITW Reagents (AppliChem GmbH, A3830,0500, Darmstadt, Germany) and was used as received. TRIS buffer, sodium hydroxide (NaOH), methanol (MeOH), hydrochloride acid (HCl), were purchased from Sigma-Aldrich Chemie GmbH, Steinheim, Germany. Distilled water was purified using a Milli-Q system (Purelab Option-Q, ELGA, High Wycombe, UK).

### 2.2. Processing PLLA Films

Piezoelectric polymer films were prepared using the following procedure, optimized in our previous study [18]. PLLA granules (1 g) were melt-pressed between two metal plates at 200 °C under a pressure of 56 kN for 3 min and immediately quenched in cold water (4 °C) (amorphous sheet). To prepare piezoelectric films, the amorphous sheet was cut into a dumbbell-shape film and uniaxially stretched with a homemade tensile stretcher to a draw ratio of 5 at a temperature above the glass transition (90 °C), using a drawing rate of 40 mm/min. Surface modification, such as alkaline etching, was performed to prepare hydrophilic films. Films were submerged overnight in a 0.04 M NaOH medium, prepared in a water/MeOH mixture (70/30 *V/V*) to cleave the ester bonds on the surface.

### 2.3. Enzymatic Degradation

The drawn films were immersed in 0.1 mg/mL proteinase K solution with pH 8.5. The enzyme solution was prepared using a 0.5 M TRIS buffer, adding HCl to adjust the pH value. 5 mL of enzyme solution containing films was maintained at 37 °C in a water bath while gently shaken. The degradation study was carried out for 10 days. Films were washed with water and left to dry before the analyses. Enzymatic activity during the degradation process was monitored using an absorbance multiplate reader (Synergy H1, BioTek, Bad Friedrichshall, Germany). A sample of 500 µL of enzymatic medium taken from the degrading sample was added to 500 µL of 1 mg/mL BSA protein and digested for 2 h. Absorbance at 290 nm was continuously measured to determine the half-time needed for protein degradation. Measurements were made in two parallel samples for enzymes with different polymer films and a bare enzyme. Further enzymatic activity was stopped by heating the sample at 90 °C. A total of 15 µL of the sample with 3 µL of added loading buffer were put on 15% polyacrylamide gel to separate the degraded BSA proteins based on size using SDS-page electrophoresis.

### 2.4. Characterization Methods

Gravimetric determination, crystallinity and orientation changes were calculated using the following equation:

(1) $$\Delta X\;\left(\%\right)=100\%\times \left(X_{t0}-X_{t}\right)/X_{t0},$$

where ΔX represents the weight changes (w), crystallinity (*X*) and orientation ratio (D) from the beginning (*t*0) to a certain time of degradation (*t*).

Orientation was determined using a Fourier-transform infrared spectrometer in attenuated total reflectance (ATR) mode (PerkinElmer Spectrum 100, Waltham MA, USA). Spectra were recorded in the 600–4000 cm^−1^ wavenumber range with a spectral resolution of 4 cm^−1^ and the accumulation of 10 spectra using a polarizer. Changes in the orientation were determined using the previous equation, where D represents the ratio between changes of the vertical (‖) and horizontal (⊥) absorbance (*A*) of the C=O peak (1756 cm^−1^) using the following equation [19,20]:

(2) $$D=A_{∥}/A_{⊥}.$$

Crystallinity was determined using a NETZSCH STA 449 (Jupiter) thermal analyzer for differential scanning calorimetry (DSC) in an Ar/O atmosphere (40/10). A total of 3–4 mg of each sample were put in platinum crucibles and heated from 40 °C to 200 °C with a 20 °C/min heating rate due to the temperature calibration under these conditions. The enthalpy of cold crystallization (∆Hc) and the enthalpy of melting (∆Hm) were determined by calculating the surface under the peak of the crystallization or melting, respectively. Bulk crystallinity was determined with the following expression:

X_c_ (%)= 100% × (∆H_m_ − ∆H_c_)/∆H_100%_, (3)

where the value for ∆H_100%_ is taken as 93.6 J/g, which is a theoretical value for 100% crystalline PLLA films in the α crystalline form [21].

Morphological changes during degradation were observed with a scanning electron microscope (JSM-7600 F, Jeol Ltd., Tokyo, Japan). To observe changes in the hydrophilic properties, methylene-blue staining was used to observe the color change due to more carboxylic groups on the surface [22]. Water wetting angles were measured using a Theta Lite contact-angle meter, Biolin Scientific.

Dynamic mechanical properties of the PLLA samples were studied under tension mode on the films in a rectangular shape (9 mm long, 3–3.5 mm wide and 0.1 mm thick). The measurements were performed with a Mettler Toledo DMA/SDTA861e. The dynamic responses were tested from 0 °C to 130 °C at the heating rate of 3 K/min. The dynamic force amplitude was 1 N, and the validity of Hooke’s law (linearity measurement) was tested on every sample to determine the displacement amplitude, which was 2–5 µm. The chosen frequency was 1 Hz. The storage modulus, loss modulus and tan δ were recorded as a function of the sample temperature. Since the measurements were made in the tension mode, the storage modulus corresponds to Young’s modulus.

The piezoelectric properties were measured according to the description in a previous article [18]. Polymer films were cut at an angle of 45° from the stretching axis, seeing that the shear stress has a maximum value when measured at this angle [23]. The measurement was made using a PiezoMeter System PM300 (Piezotest Pte. Ltd., International Plaza, Singapore), which was adapted for thin-film d_31_ measurements. First, gold electrodes were sputtered on both surfaces of the polymer film. Then the film was clamped on both sides to stretch the film (frequency of 110 Hz and force 0.15–0.5 N). The voltage was measured with a voltmeter (Tenma multimeter) over a reference capacitor of 1000 pF. The piezoelectric coefficients g_14_ and d_14_ were calculated.

## 3. Results

**Figure 1:**
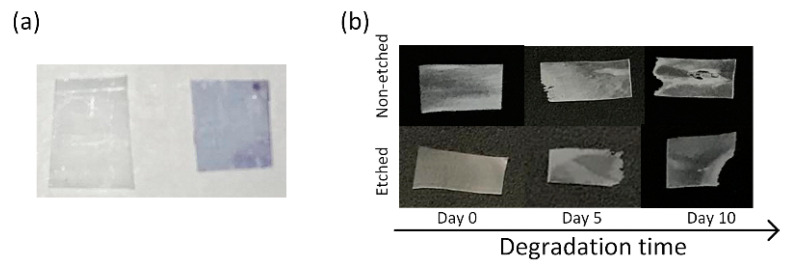
(**a**) Methylene blue staining of non-etched (**left**) and etched (**right**) PLLA films; (**b**) samples of enzymatic degradation of the non-etched (**top**) and etched (**bottom**) films after 0, 5 and 10 days.

PLLA films were made with a hydrophilic surface using alkali etching to improve their water wettability, which is favored for interactions with cells [24]. Before being applied for the degradation study, the stretched, oriented, and etched films remained stained after immersion in the methylene-blue solution, indicating a larger amount of carboxylic end groups on the surface (Figure 1a). Degradation was performed using a proteinase-K-buffered solution under simulated physiological conditions (gently shaking at 37 °C). After aging for 5 and 10 days in the enzyme solution, the polymeric films had macroscopically observable damage (Figure 1b). Normally, for PLLA polymers, proteinase K acts as a hydrolysis catalyzer, and degradation follows the surface mechanism [15]. For the case of non-etched, hydrophobic films, we initially observed the occurrence of surface erosion, along with some evidence of bulk erosion induced by autocatalysis (day 10). This result was more following the available literature on hydrophobic PLLA film degradation without any enzyme present [17]. However, in the case of more hydrophilic PLLA films, it was observed that the films were macroscopically more compact, without signs of bulk erosion, indicating a contribution of the surface modification to the degradation process and the following mechanism (Figure 1b).

### 3.1. Enzymatic Activity

**Figure 2:**
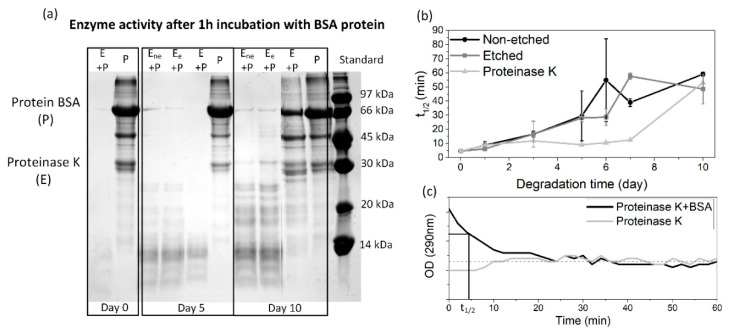
(**a**) SDS-page results for the enzymatic activity of the initial solution and after 5 and 10 days of degradation for enzymes in non-etched (Ene), etched (Ee) and control solutions (E), where activity was verified with degradation of the BSA protein (P); (**b**) calculated half time needed for BSA protein degradation for enzymes in non-etched, etched and control solutions, determined from measured optical density at 290 nm for each solution, where graph (**c**) presents the absorbance measurements for initial proteinase K solution with a marked half time of total degradation (t_½_).

Since all of the polymeric films were degraded in the same solution for the whole 10 day period, the enzyme activity was periodically monitored to follow the progress of degradation. By lowering the pH value, as a result of the release of lactic acid residues into the medium during the degradation progress, the activity of the proteinase K was also expected to decrease. The detected degradation products (based on an SDS-page test) (Figure 2a) and the half time of the enzyme’s degradation activity (Figure 2b) show a lowering of the activity along with the film’s degradation. The enzyme activity was also decreased in a reference enzyme solution (without films); however, this drop was more pronounced when films were present. Figure 2c presents the activity of the initial enzyme solution with the reference BSA protein, observed with a continuous absorbance measurement, from where the half time of the total protein degradation was determined. After 10 days of degradation, the enzyme activity is significantly reduced compared to day 1 (from 5 to 50 min), even without the films being present. This was expected since no fresh enzymes were added during the degradation. Despite the observed decreases, it should be noted that the enzyme was active for the whole period during which the degradation progress was observed. Accordingly, all the observed changes in the aged PLLA films could be assigned to the process of degradation.

### 3.2. Structural Changes (Crystallinity and Orientation)

After 10 days of degradation, the weight losses, indicating the progress of the degradation, were similar for both the etched (35%) and non-etched (37%) films. Additionally, the drops in the pH, directly correlated with the degradation process, were comparable for the etched and non-etched films (from 8.5 to 4.6) since the equally released lactic acid residues lower the initial pH value. The initial hydrophilicity was changed only on the surface of the films, which could affect the location of the degradation; however, the total progress of the degradation remained the same.

**Figure 3:**
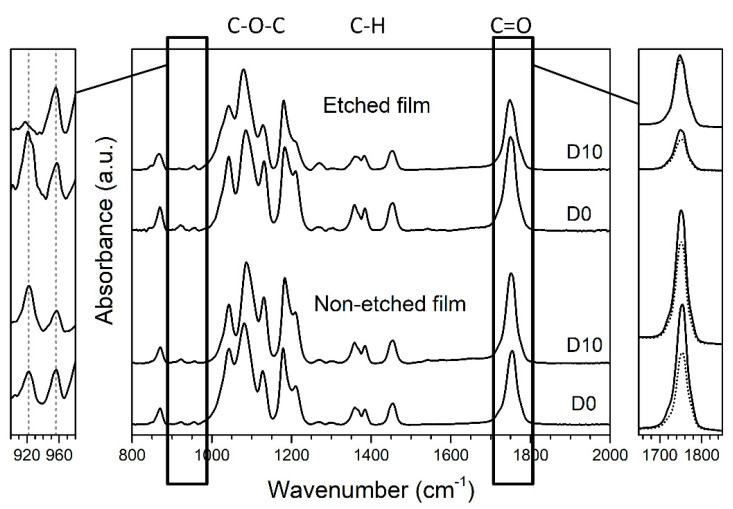
ATR FT-IR spectra (normalized to maximum peak absorbance) for non-etched and etched PLLA films (**middle**), degraded for 10 days in enzymatic solution (D10), compared to initial PLLA film (D0). On the (**left**), an enlarged, normalized spectrum between 900 and 980 cm^−1^ is presented to observe changes in the intensity of peaks that are specific for more crystalline (921 cm^−1^) or more amorphous (956 cm^−1^) films. On the (**right**), a polarizer was used to determine the changes in orientation of the C=O peak, comparing the intensities of horizontal (solid line) and vertical (dotted line) orientations without normalization of the spectra.

The main differences in the degradation of the etched and non-etched films were the crystallinity and orientation. These properties were detected by comparing the FTIR spectra of the enzymatically degraded films with the spectra of the film reference (corresponding to films aged in a medium without the enzyme present), as presented in Figure 3. Typical changes in the surface crystallinity are observed closely at normalized spectra in the wavelength range 900–980 cm^−1^. Following previous studies [16,18], the increase in the intensity of the peak at 922 cm^−1^ represents an increase in the crystallinity of the PLLA, which we also observed in the case of the non-etched (hydrophobic) films (Figure 3, left).

Orientation for the polymeric chains within the film was confirmed based on an observable peak at 1755 cm^−1^ that corresponds to the C=O stretching in the ester carbonyl group. As noted earlier, high anisotropy is obtained for oriented polymeric films when comparing the vertical and horizontal positions [19]. For our oriented PLLA films, the intensities of the C=O and C–O–C peaks are enhanced in the drawing direction (horizontal) compared to the perpendicular direction (vertical) and shifted to slightly lower values (Figure 3, right). Comparable changes in the C=O and C–O–C peaks were also observed by T. Nobeshima et al. [20]. Regarding the influence of the water wettability, the orientation was decreased for both the hydrophobic non-etched and hydrophilic etched films during degradation (Table 1 and Figure 3).

**Figure 4:**
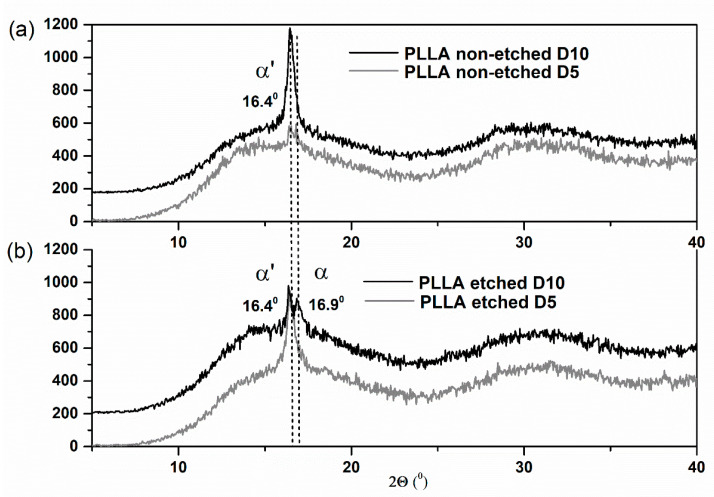
XRD patterns of hydrophobic non-etched (**a**) and hydrophilic etched (**b**) PLLA films obtained after 5 and 10 days of degradation by proteinase K (labeled with D5 and D10).

Similar structural changes were revealed during the XRD study (Figure 4). As with the FTIR analysis, the XRD results clearly indicate the large increase in crystallinity of the non-etched samples. The polymer is in the α′ phase since the specific (200)/(110) peak appears at the 2-theta position lower than 16.6°, following a previous report [25]. After 10 days, the additional, more ordered α phase is observed only for the etched film, detected as an additional XRD peak at 16.9° (Figure 4b).

**Figure 5:**
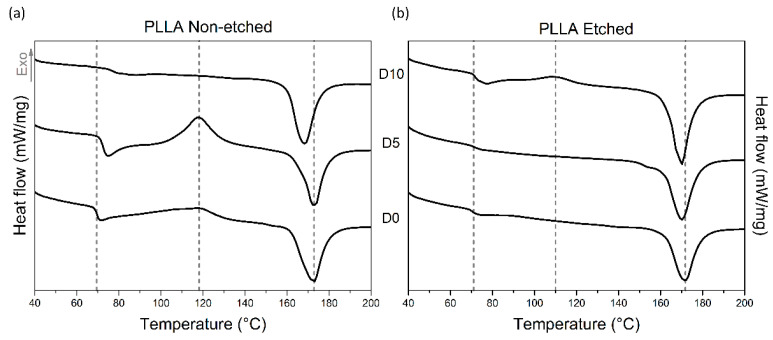
DSC curves of hydrophobic non-etched (**a**) and hydrophilic etched (**b**) PLLA films obtained after different periods of degradation by proteinase K, labeled by D0, D5 and D10.

Investigating the thermal properties of PLLA films also revealed different responses to enzyme-catalyzed degradation for the etched and non-etched samples (Table 1, Table 2 and Figure 5). For the etched samples, the melting peaks are broad for all the measured samples, indicating the presence of both crystalline forms: α′ and α. Degradation promotes recrystallization (α′ to α) since they are both detected in the final degradation stage, as seen in the XRD data. The crystallinity of the etched films, measured using DSC, is initially high (since etching removes part of the amorphous regions), and it was not changed much during the degradation. However, for non-etched samples, even though it starts at a lower value, the crystallinity increased significantly after 10 days of degradation, as also observed from the FTIR and XRD analyses. While the glass and melting temperatures (T_g_ and T_m_) were shifted for non-etched films during degradation, similar changes were not observed in the etched films. H. Tsuji et al. also observed increased T_g_ values for the oriented and un-oriented films; therefore, T_g_ changes respond to the degradation process, like changes in the highly ordered structure, such as crystallinity or orientation [15].

### 3.3. Mechanical Properties

DMA measurements were performed for degraded samples to determine the changes in the mechanical properties. Since the non-etched films were very torn and porous after 10 days of degradation, measurements were not feasible. Therefore, the mechanical properties were measured only for the 5 day degradation period. On the other hand, in the case of the hydrophilic etched films, the compactness of the film was less damaged by degradation, so the mechanical measurements were normally made after 5 and 10 days of degradation.

**Figure 6:**
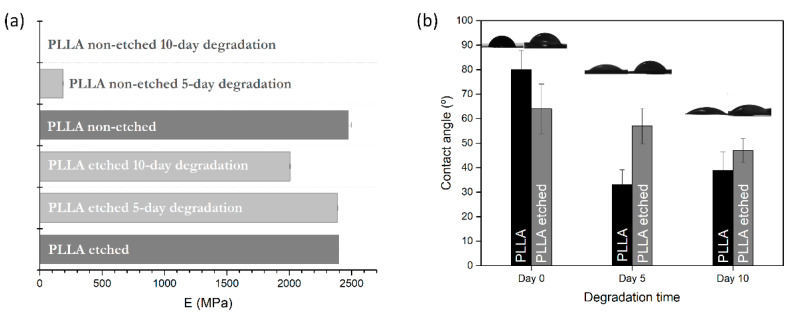
(**a**) changes in Young’s modulus (at 23 °C) and (**b**) changes in wetting angle for water drop on polymer, for non-etched and etched samples after 0, 5 and 10 days of degradation.

Both etched and non-etched PLLA films have the same initial Young’s modulus measured at 23 °C (2.4 GPa), comparable to values observed in the literature for PLA polymer [26], which confirms that alkali etching was performed only on the surface without compromising the inner parts (Figure 6a). After the enzymatic degradation, the values are significantly lowered for the non-etched samples, even after 5 days of degradation, where the modulus is lowered by 92% (190 MPa), while it is only 1% for the etched samples (Figure 6a). As the degradation induces swelling and increased porosity in the bulk of the hydrophobic films (where degradation occurs), they cause a drop in the mechanical properties (observed through a drop in the storage modulus). These changes are not so pronounced in the case of the etched films before and after degradation since the dominant degradation events are taking place at the surface. In the case of the etched, hydrophilic PLLA films, Young’s modulus drop occurs very slowly, only 16% for a degradation time of 10 days, clearly showing the lack of dominant bulk-erosion effects observed in non-etched films.

### 3.4. Wetting-Angle Changes

Contact-angle measurements were made for pristine films and the films obtained after degradation. Interesting changes were observed for the non-etched films, where the wetting angle decreases more drastically than for the etched sample after 5 and 10 days of degradation (Figure 6b).

Alkali etching improved the hydrophilicity of the polymer surface by 25%, which resulted in more carboxylic end groups on the surface. During the degradation, a small lowering (−20%) of the initial wetting angle was observed in the etched films. The change could be associated with the change in the roughness observed in the SEM images for the etched films. On the other hand, due to the significant increase in the roughness and porosity of the non-etched hydrophobic films after degradation, their wetting angles decreased by 50%.

### 3.5. Piezoelectric Properties

Piezoelectric properties are highly dependent on the polymeric structure, the orientation of the polymer chains and the degree of crystallinity. Therefore, it was expected that the previously observed changes would affect them. According to those changes, proteinase-K-induced changes in the piezoelectric properties were lowered to 68% of the initial values for the non-etched sample and 50% for the etched polymer films (Table 1) after 5 days of aging. Further measurements after 10 days of degradation could not be performed since the degradation, with a mechanically unstable and porous film over the whole surface, did not allow measurements using the applied method.

### 3.6. Morphological Properties

**Figure 7:**
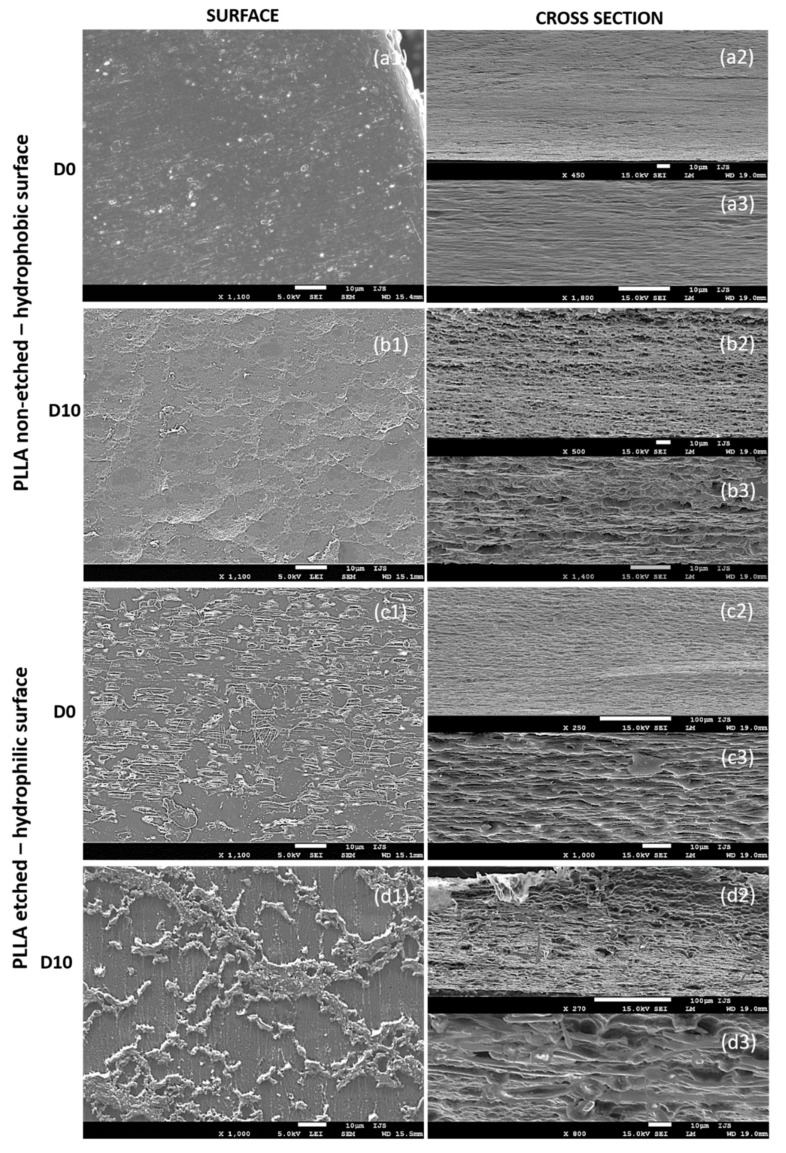
SEM images showing surfaces (1) and cross-sections (2, 3) of PLLA films with non-etched hydrophobic (**a1**–**a3**,**b1**–**b3**) and etched hydrophilic (**c1**–**c3**,**d1**–**d3**) surfaces before (D0) and after (D10) degradation with proteinase K.

After 10 days of degradation for the non-etched hydrophobic films, the main changes were observed in the cross-sections that reveal their inner (bulk) parts (Figure 7(a2,a3,b2,b3)). The initially dense and smooth layered structure in the cross-section of the starting films before degradation (Figure 7(a2,a3)) turned into a porous, sphere-like structure obtained after the degradation progressed (Figure 7(b2,b3)). Both surfaces of the films, before and after the degradation (Figure 7(a1,b1)), are generally smooth, with a bubble structure observed on top of the degraded samples (Figure 7(b1)), indicating degradation events inside the film’s bulk, such as progressive and intensive water swelling.

Due to alkali etching, the hydrophilic films initially had a rougher surface with visible amorphous islands having short-edged chains (Figure 7(c1)). Such a structure is ideal for enzymatic degradation. As the degradation progressed, the roughness of the surface changed (Figure 7(d1)), indicating surface-erosion events. The inner parts of the films (observed in cross-sections, Figure 7(c2,c3,d2,d3)) did not change significantly and showed signs of delayed water swelling.

## 4. Discussion

Piezoelectric PLLA films were designed to optimize their applicability in electrostimulation, such as promoting mammalian cell growth. For the occurrence of effective electrical stimulation, film-cell contact is crucial, and surface properties are important. Therefore, to improve its properties for intended medical use and to ensure better cell affinity, hydrophilicity was improved by 25% using alkali etching of the polymer film, which resulted in more carboxylic end groups on the surface. The idea of the current study was to investigate the structural, mechanical, electric and surface properties of piezoelectric PLLA, with a hydrophobic and hydrophilic surface, in the presence of proteinase K as a degrading enzyme and a model of in vitro inflammation. This study is important since it can predict or explain further interactions of the surface-modified PLLA with living surroundings.

PLLA is a hydrophobic polymer that in general degrades through a hydrolysis reaction that occurs inside the bulk, driven by the swelling mechanism [14]. In the case of enzyme-driven degradation, when the enzyme proteinase K is present, surface erosion is reported as the main mechanism of degradation [15,16]. The reason is associated with the limited intake of the enzyme inside a polymer film.

According to our results, the structural, morphological, mechanical and piezoelectric changes obtained during the proteinase-K-induced degradation in PLLA films, two different degradation mechanisms are revealed due to the surface modification. In the case of the non-etched hydrophobic films, changes were mainly taking place in the polymer bulk. Due to the smooth surface and the strained, oriented chains observed with the electron microscope, enzyme–polymer contact is less probable; therefore, surface degradation is limited, and water diffusion inside the bulk is faster. Swelling occurs, which allows the enzymes to enter inside, meaning that bulk erosion is preferential. Enzymes cleave the tied and free amorphous parts from inside the bulk since proteinase K cannot degrade crystalline parts, resulting in increased overall crystallinity. Similar observations were made by Rangari et al., who also reported a slight increase in the crystallinity for oriented PLLA films due to the enzyme-catalyzed degradation of amorphous parts [16] but at the surface. The observed changes in crystallinity for the non-etched samples can be explained by the degradation of the free end and tie amorphous parts and possible accumulation of the cleaved crystalline parts trapped inside the film (increased crystallinity for day 10), as the degradation occurs there. However, the loss of crystallinity observed for day 5 can be associated with chain relaxation during the swelling mechanism. In both cases, for etched and non-etched films, the orientation of the polymeric films is lowered, which is expected since the swelling of the polymer in a water solution is inevitable after some time, making reorganization and mobility of the chains possible.

When observing etched films, their surface is hydrophilic with more amorphous end chains directly exposed to the outer water surroundings with the proteinase K enzyme. During the degradation, the film changes predominantly at the surface-with-surface erosion as the preferred mechanism. Therefore, only small changes are observed for bulk crystallinity and mechanical properties. Since the dense film is under amorphous clusters (as observed 7d1), the accumulation of crystalline residues is also less likely, and the loss of crystallinity is observed only on the surface.

F. Iñiguez-Franco et al. showed that increasing the hydrophilic properties of PLLA film using a chain extender, which incorporates more hydrophilic chain ends to form a branched structure, can result in hydrogen bonding between them and prevent the diffusion of water molecules inside the polymer matrix to start the bulk process [27]. This explanation can be used to understand the changes we observed with the hydrophilic samples. In our case, the diffusion of water inside the films was slower, and enzyme travel inside the bulk was prevented. Therefore, enzymatically catalyzed hydrolysis occurs only at the surface (FTIR-921 cm^−1^ peak drop), inducing surface erosion. The loss of crystallinity for the etched sample occurred due to cleavage of the tie amorphous chain parts at the surface, releasing crystalline residues into the solution. Similar phenomena during degradation were previously explained by H. Tsuji et al. [15] on surface-eroded hydrophobic PLLA films. We also observed lowering the temperature of cold crystallization for the etched sample compared to non-etched films, which implies better orientation of the amorphous chains since the presence of oriented amorphous chains induces crystallization at a lower temperature. An interesting partial change from the α′ to the α structural phase was observed only for etched films and further indicates the difference in degradation induced by surface wettability. This phenomenon could be explained by the recrystallization of polymer chains due to annealing in the medium at 37 °C since water can act as a plasticizer for PLLA. This was explained by H. Tsuji et al. as a possible event during degradation [15]. Since most of the degradation events in our etched films occur on the surface, this should also be the case for recrystallization.

The use of PLLA films for piezo-stimulation depends on their mechanical properties. PLLA is a semi-crystalline polymer; therefore, its properties depend largely on its crystalline phase. However, the balance of crystalline and amorphous regions is desirable since it enables the elasticity required for deformation in ultrasound that generates the voltage needed for promoting cell growth during stimulation. A difference in mechanical properties also implied a bulk-degradation event for non-etched and a surface-erosion event for the etched films during degradation. A huge loss of modulus, observed for non-etched films, could be connected to the swelling of the polymer film, as observed before [28], which could indicate that the degradation process was greater than that, which was evident with the weight loss. Due to swelling, there is an internal cleavage of the molecular bonds inside the film, which affects their compactness and strength, promoting degradation without the evident weight loss [1]. A similar situation was not detected in the etched films as their degradation took place at the surface.

Piezoelectricity in PLLA is a consequence of oriented and crystalline polymer chains inside the film [11]. A simple method like uniaxial drawing was used for preparing piezoelectric PLLA films. The drawing process above T_g_ aligns the chain molecules in the same direction in the entire film, inducing structural changes, such as crystallinity, due to the strain-induced crystallization [18]. Compared to the literature (10pC/N) [29], smaller values for d_14_ were obtained for our films (4–4.6 pC/N); however, negligible changes were observed after the surface modification with etching to improve the hydrophilic properties of the film (results presented in a previous article [18]). It is very important that the films retained their piezoelectric properties despite all the structural and mechanical changes during degradation. Changes in the piezoelectric properties after the degradation were observed for the first time. The preservation of piezoelectric properties is a consequence of changes in crystallinity and orientation. Bulk changes are not so pronounced in hydrophilic films, where most of the degradation events and the consequent modifications are taking place at the surface. However, the detected recrystallization, loss of surface crystallinity and relaxing orientation cause the loss of half of the initial piezoelectricity (50.4%, Table 1). In hydrophobic films, most of the degradation events and the following changes occur inside the films. Although there is a decrease in the orientation and an observed increase in the porosity, a smaller drop in the piezoelectric properties (68.8% of initial value remain, Table 1) was observed, possibly due to an increase in the crystallinity and the reorientation of fibers after drying the sample. However, our estimation is for the non-etched film to have a lower piezoelectric value during the degradation since the polymer is swollen from the start, compared to the etched sample, where the swelling was not observed after 5 days of degradation.

A combination of a hydrophilic surface with gradual changes to the films during degradation that maintains their piezoelectric properties is the optimal design for polymeric films initially developed for interactions with mammalian cells. With such a design, we could expect that the high affinity of cells for hydrophilic surfaces will enable close contact between the cells and films and provide the effective electrostimulation required for applying piezo-films in regeneration processes, particularly in wound healing.

## 5. Conclusions

Proteinase K can induce both bulk and surface degradation in PLLA films. The surface properties of the polymer films play a significant role in their degradation. As shown in the present study, their change is a powerful tool and makes it possible to change where the degradation occurs and defines the dominant degradation mechanism. If films are hydrophilic, enzyme-driven degradation occurs at the film surface, where they degrade the accumulated amorphous areas. On the other hand, if they are hydrophobic, water uptake and polymer swelling make it possible to transfer the enzyme to the bulk where the degradation occurs. When the surface chemistry is well balanced by degradation progress, so the mechanical, structural and piezoelectric changes to the films occur gradually, and very effective interactions with the cells are expected. The observed correlations are very important for further predictions during the interactions of piezoelectric PLLA films with living surroundings, particularly during electro-stimulated regeneration and wound healing, where the gradual loss of piezoelectric properties is useful for following the tissue regeneration.

## Figures and Tables

**Table 1:** Summarized properties of degraded PLLA films over 10 days in solution with proteinase K.

Up to 10 Days of Degradation	PLLA Non-Etched	PLLA-Etched
**pH change**	From 8.5 to 4.6	From 8.5 to 4.6
**Weight loss**	37 wt %	35 wt %
**Crystallinity change (DSC)**	+27%	+5%
**Orientation change** **(FT-IR: 1750 cm^−1^)**	−24% of the initial ratio	−18% of the initial ratio
**Piezo change**	**After 5 days:**	**After 5 days:**
**d_14_** **g_14_**	2.68 pC/N (68.8% of initial) 0.092 Vm/N	2.32 pC/N (50.4% of initial) 0.066 Vm/N
	**After 10 days**	**After 10 days**
**d_14_** **g_14_**	Non-measurable with applied method	Non-measurable with applied method

**Table 2:** DSC data for etched and non-etched PLLA films for different periods of degradation.

Up to 10 Days of Degradation	T_g_	ΔH cryst. (J/g)	T_c_	ΔH melt (J/g)	T_m_	Crystallinity %
**PLLA-non-etched**	69 °C	21	117 °C	51	173 °C	31
**PLLA-non-etched D5**	72 °C	35	118 °C	50	173 °C	16
**PLLA-non-etched D10**	75 °C	/	/	55	167 °C	58
**PLLA-etched**	71 °C	/	/	49	172 °C	52
**PLLA-etched D5**	72 °C	/	/	57	170 °C	61
**PLLA-etched D10**	72 °C	5	109 °C	59	170 °C	57

## Data Availability

The data presented in this study are available on request from the corresponding author.
